# Supplementary material for: Observation of elastic spin with chiral meta-sources
Source: Nat Commun. 2021 Nov 29;12:6954. doi: 10.1038/s41467-021-27254-z (PMC8630157; doi:10.1038/s41467-021-27254-z)
Supplement: Supplementary file 1 — Supplementary Information [file 41467_2021_27254_MOESM1_ESM.pdf]

Supplementary Information-Observation of elastic spin with chiral meta-sources

CONTENTS

Supplementary note. A. **Sample fabrication** 1

Supplementary note. B. **Experimental measurements** 1

Supplementary note. C. **Observation of elastic spin for A_0 wave upon spin-down source** 1

Supplementary note. D. **The selection of 5-cycled tone burst pulse signal for wave generation** 2

Supplementary note. E. **Factors affecting the sub-wavelength chiral source** 3

Supplementary note. A. SAMPLE FABRICATION

The experimental samples are built up on conventional 6061-T6 aluminum plates. Supplementary Fig. 1(a) shows the photograph of A_0 wave sample that consists a plate with size 180 cm $\times$ 6 cm $\times$ 0.8 cm, in the x, y and z axes, respectively. x axis is along the propagating direction, y axis is along thickness direction, whereas the z axis is vertical to x-y plane. The plate has thickness 6 cm rather than 0.8 cm. So that the x axis changes from -90 cm to 90 cm, and y axis occupies 0 $\sim$ 6 cm. The surfaces supporting Lamb waves are x-z surfaces at both y = 0 and y = 6 cm. Regarding Rayleigh wave sample, the plate thickness becomes 120 cm along y axis, being about 8 times the largest Rayleigh wavelength of $\sim$ 15.4 cm in this work, so as to mimic the semi-infinite condition for Rayleigh wave.

The chiral elastic source, as shown in Supplementary Fig. 1(b), is installed around center at (x, y) = (0, 1.2 cm). The chiral source constitutes four sub-sources in square lattice with the lattice distance $d = 12$ mm. For each of them, an aluminum rod (green) whose radii $r = 2.25$ mm is firstly fixed by epoxy adhesive (red) to the air hole perforated through aluminum plate (gray), and then two lead zirconate titanate piezoelectric ceramic (PZT) rings (blue) are symmetrically glued by epoxy adhesive to the two laterals of aluminum rod. The PZT rings used here are made of PZT-8 with the outer diameter 10 mm, inner diameter 5 mm, and thickness 1 mm. The distance from top (bottom) surface of an aluminum rod to closest surface of aluminum plate is l, the distance from top (bottom) surface of an aluminum rod to closest surface of PZT ring is h, the radii of holes drilled in aluminum block is r_1 = 2.5 mm, the radii of aluminum rods is r = 2.25 mm, the length of aluminum plate along z axis is e = 8 mm, and the distance between the meta-source center and x-z surface at y = 0 is L = 12 mm. In this way, electrical signals generated upon the PZT rings can transpose forces to the aluminum rods into breathing vibration and then generates elastic waves in the substrate solid structures. However, due to the efficient generation of breathing mode, the aluminum rod cannot be long along the z axis, and consequently, the plate size is chosen to be 0.8 cm for both A_0 and Rayleigh waves.

Supplementary note. B. EXPERIMENTAL MEASUREMENTS

The 5-cycled tone burst electrical signals with central frequency f_c are issued from the function generator (RIGOL DG1032z) and amplified by the power amplifier (Aigtek ATA-2022H). Concerning the measurement, the laser Droppler vibrometer (LDV, Polytec vibrometer OFV 2570) is used to record the out-of-plane displacement u_y at any position on the x-z surface. Each measurement comes from the averaging over 256 scans digitized by an oscilloscope (DPO 4102B) at sampling frequency of 100 MHz. This measurement allows for a good signal-to-noise (S/N) ratio.

To obtain SAM of elastic wave along x-z surface, small V-shape grooves with size about 5 mm $\times$ 5 mm were drilled as shown in Supplementary Fig. 1(c). Each groove has two faces with side length 5 mm, and it brings almost no influence on wave transmission since the size is much smaller than the wavelength of elastic wave in this work. In experiment, we measure only the out-of-plane displacementson the two faces of each groove. Let us label the out-of-plane displacement as u_2 when measured at the right face and as u_1 at the left face. Then, we calculate the u_x and u_y on the x-z surface for an effective point in between each groove. For simplicity, the effective point is marked as the central point of each groove on x-z surface, to be x = ±45 cm in the end. In detail, for the small grooves on front x-z surface y = 0, the u_x and u_y can be obtained as u_x= $u_1cos(\theta_1) - u_2cos(\theta_2)$ and $u_y = -u_1sin(\theta_1) - u_2sin(\theta_2)$, while for grooves on back x-z surface y = 6 cm only in A_0 wave system, the $u_x = u_1cos(\theta_1) - u_2cos(\theta_2)$ and $u_y = u_1sin(\theta_1) + u_2sin(\theta_2)$. The value of angle θ_1 and θ_2 is 45°.

Supplementary note. C. OBSERVATION OF ELASTIC SPIN FOR A_0 WAVE UPON SPIN-DOWN SOURCE

The spin-down source with central frequency f_c = 14 kHz was generated. Then, we measured the u_y of both the left-going ($x < 0$) and right-going waves ($x > 0$) every 1 cm, on the x-z surfaces at y = 0 and y = 6 cm, respectively. Supplementary Fig. 2(a) shows the normalized 2D-FFT components of u_y recorded at back x-z surface y = 6 cm, magnifying a

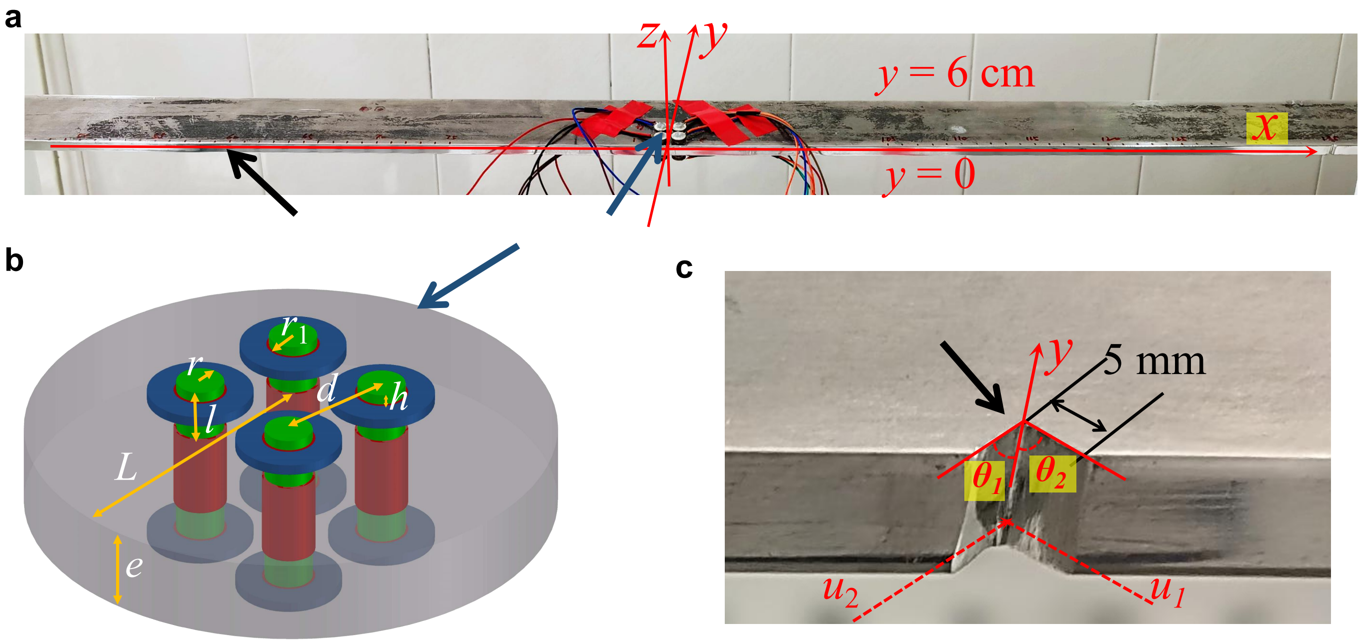

Supplementary Figure 1. **a** Photograph of experimental sample for A_0 wave where a chiral elastic source installed around point at (x, y) = (0, 1.2 cm). **b** Schematic of chiral elastic source built up by inserting four group of PZT rings with distance $d = 12$ mm. For each group, an aluminum rod (green) with radii $r = 2.25$mm is firstly fixed by epoxy adhesive (red) to the air hole perforated through the aluminum slab (gray), and then two PZT rings (blue) are symmetrically glued by epoxy adhesive to the two laterals of the aluminum rod, the distance from top (bottom) surface of an aluminum rod to closest surface of aluminum plate l, the distance from top (bottom) surface of an aluminum rod to closest surface of PZT ring h, the radii of holes drilled in aluminum block r_1 = 2.5 mm, the length of aluminum plate along z axis e = 8 mm, and the the distance L between the center of meta-source and x-z surface at y = 0. **c** Photograph of V-shape groove with side length 5 mm which is much smaller than the wavelength in our systems, and the measurement of the out-of-plane displacement on two faces of grooves.

primary spot well located on the right A_0 branch, i.e. the domination of right-going A_0 wave over the left-going one. At the same time, the components of S_0 wave are minor. The FFT component of u_y of each point measured on back x-z surface y = 6 cm, as shown in Supplementary Fig. 2(b), also shows the prior generation of rightward A_0 wave, being consistent with the numerical results in Supplementary Fig. 2(c). Supplementary Fig. 2(d) shows the experimental profiles of u_x and u_y at the point x = 45 cm and y = 6 cm, which leads to the anti-clockwise rotation of $\boldsymbol{u}$ and the positive $\boldsymbol{S}$ for the rightward A_0 wave. The results obtained on front x-z surface y = 0, shown in Supplementary Fig. 2(e)-(g), feature the same rightward A_0 wave, but demonstrate the negative SAM at x = 45 cm and y = 0, being just opposite to the one on x-z surface y = 6 cm. Importantly, Supplementary Fig. 3 shows that the chiral selective routing of A_0 wave can occur in a broad frequency range whenever using the elastic spin-up or spin-down source.

Supplementary note. D. THE SELECTION OF 5-CYCLED TONE BURST PULSE SIGNAL FOR WAVE GENERATION

The choice of 5-cycled burst pulse signal comes from the experimental limitations during characterization of displacement field and phase. First and experimentally, wave reflection that occurs on solid-air interface is hard to be eliminated due to the difficulty of realizing an absorbing boundary for elastic wave. Secondly, we cannot elaborate a sample as infinite as possible so that wave reflection is almost unavoidable. In this context, an usual way is adopting pulse signals with short exciting time that can help people distinguish the initial wave and the reflected wave within finite sized sample. Without losing generality, we adopt the widely used tone burst signal. Supplementary Fig. 4 shows the initial elastic wave package and the well separated reflective wave package when the cycle number of burst signal is 5. This cycle number of tone burst signal can be tuned. But one have to keep in mind that, when the cycle number is quite large, e.g. 10, the duration time of initial wave package and the reflective wave package can overlap with each other, leading to the interference between waves along opposite direction. When the cycle number is too small, e.g. 1, the operation width of pulse signal is quite large in comparison the central frequency f_c, and the chiral source loses efficiency since the phase difference between signals applied on each rod are tuned according to the central frequency f_c. Based on this consideration, we adopt the widely used 5-cycled tone burst signal as F = 1 × rect(t) × [1 - cos $(2\pi f_c t/5)$]×sin$(2\pi f_c t)$ where rect(t) is a rectangular pulse with limit $0 \leq t \leq 5/f_c$.

This burst signal is also featured by the smooth transition of voltage from zero charge to peak value, which can prohibit

the unexpected rod vibrations caused by sharp voltage variation when using other pulse envelopes, e.g. rectangular pulse. Lastly, when one reduces the pulse duration or increases repetition number in 1 s, the initial wave package can overlap with the reflective wave package, leading to unnecessary wave interference. On the other hand, we need quite long time to ensure the large signal to noise ratio when increasing the pulse duration time to be extremely large. In the end, our pulse duration time is set 100 ms.

Supplementary note. E. FACTORS AFFECTING THE SUB-WAVELENGTH CHIRAL SOURCE

In simulation, one can use the polarized point source located on x-z surface to demonstrate the spin-momentum locking effect. However, it is difficult to achieve this ideal polarized point source in solid aluminum blocks. To mimic such point source, the meta-source made of multi-components with sub-wavelength size are constructed with several important geometrical parameters r_1, d, l, h and L, as shown in Supplementary Fig. 1(b). These parameters shall be small in comparison to operation wavelength to allow the meta-source as equivalent of polarized point source. However, there are many practical factors to be considered, e.g., the size of PZT disk, the input signal voltage and the measuring capacity of LDV.

In experiment, r_1 is set equal to the inner radius of PZT disk as r_1 = 2.5 mm. Notice that the PZT disk cannot be too small to generate elastic wave in solid bulk. After that, we determine the lattice constant d to be as small as possible during the hand-made installation of rods and PZT disks as d = 12 mm. The L is determined in a similar way to be L = 12 mm to allow the meta-source to be very close to the free x-z surface at $y = 0$. In such a way, the smallest operation wavelength (and therefore upper frequency) limit relates principally to d, e.g. $d \leq \sim \lambda/3$ here for Rayleigh wave, apart from other factors such as properties of PZT disks.

To show more details, we turn to the 3D structure similar as our sample for Rayleigh wave. In simulation, we install identical aluminum rods with radii r = 2.25 mm into four holes drilled in aluminum plate without PZT disk. The length of rods and position of applied loads represented by l and h are essential parameters. Supplementary Fig. 5(a) shows that the large length of l can arise the instability of meta-source, while Supplementary Fig. 5(b) shows that the unidirectional propagation is more stable when h is relatively large. The instability can be simply understood by the mismatch of breathing vibrations at the loaded areas and the vibrations in the middle of rods when l is long or h is too short. Then, we symmetrically install PZT rings on rods with l = 3 mm and h = 0. As compared to the counterpart without PZT rings, i.e. the black dashed line in Supplementary Fig. 5(b), the R/L ratio reduces obviously in Supplementary Fig. 5(c) in broad frequency range. Lastly, we must admit that the pulse generated by each rod can be different after the long chain from PZT, epoxy adhesive, aluminum rod, epoxy adhesive and aluminum bulk, so that the chiral source loses some efficiency. To show the influence of this point, we tune the amplitude of voltage applied to PZT rings, e.g. those installed on rod 3, to represent the difference on rods caused by install errors or imperfect voltage load on each rod. Supplementary Fig. 5(c) shows that the unidirectional propagation is a little weakened as the voltage on PZT pair of rod 3 is 1.4 or 1.8 times the rest other ones. Nevertheless, we still get obvious unidirectional propagation in our experimental Rayleigh-Lamb wave systems whereby the $l \sim$4.7 mm and h is $\sim$2 mm, while the unidirectional direction can be ameliorated in future by modifying the geometrical parameters of meta-source or the installation process.

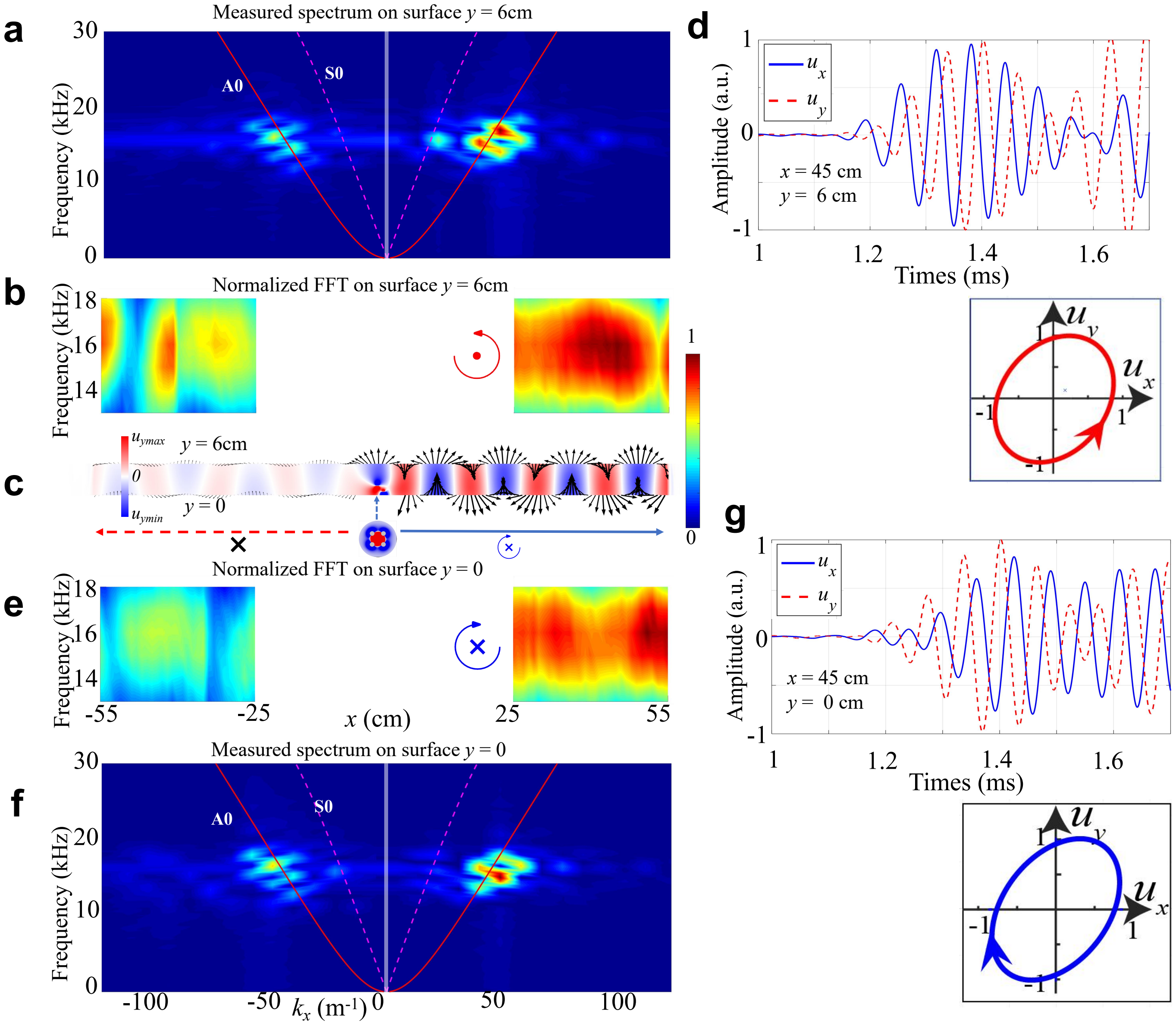

Supplementary Figure 2. Experimental observation of spin for spin-dependent chiral A_0 wave upon the spin-down source at central excitation frequency f_c = 14 kHz. **(a)** Normalized 2D-FFT component of measured signals on back surface at $y = 6$ cm. Red solid and magenta dash lines present the A_0 and S_0 branches, respectively. Hot spot spreads well over the right branch of A_0 wave. **(b)** Normalized FFT of u_y measured at each position along $x = -55 \sim -25$ cm and $x = 25 \sim 55$ cm every 1 cm on back surface $y = 6$ cm. FFT components shows the rightward wave propagation over leftward one. The rotation of displacement polarization $\boldsymbol{u}$ is anti-clockwise to give the positive SAM at rightward route. **(c)** Simulated A_0 wave propagation with SAM opposite on two surfaces of aluminum plate. **(d)** Time evolution of u_x and u_y at x = 45 cm and y = 6 cm, featuring the anti-clockwise rotation of displacement polarization $\boldsymbol{u}$ for the right-going A_0 wave. **(e)** Normalized FFT of u_y measured along $x = -55 \sim -25$ cm and $x = 25 \sim 55$ cm every 1 cm on front surface $y = 0$. The FFT components are much larger at right side, but the rotation of displacement polarization $\boldsymbol{u}$ is clockwise for the right-going waves. **(f)** Normalized 2D-FFT component of u_y on front surface $y = 0$. The A_0 wave amplitude is much larger at right branch. **(g)** The experimental profiles of u_x and u_y at x = 45 cm and y = 0, and the related clockwise rotation of displacement polarization $\boldsymbol{u}$ for the right-going A_0 wave.

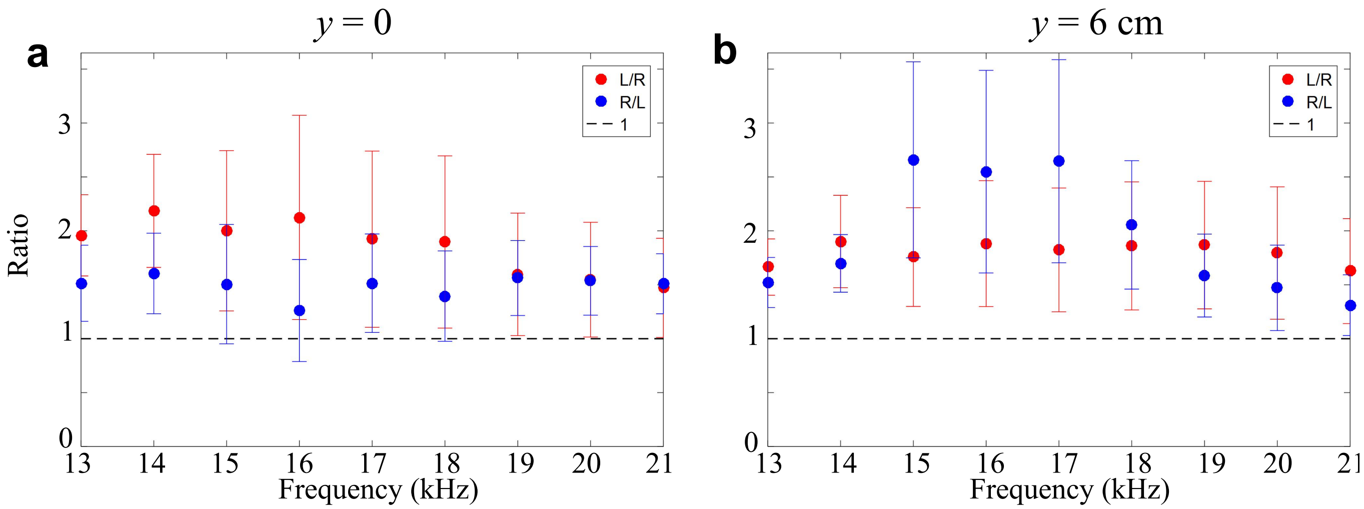

Supplementary Figure 3. Average ratios of measured $|u_y|$ of A_0 wave on x-z surfaces at (a) y = 0 and (b) y = 6 cm, within the x = -55 $\sim$ -25 cm and x = 25 $\sim$ 55 cm. In both figures, the red dots are for the $|u_{y(x<0)}/u_{y(x>0)}|$ when using spin-up source while the blue dots stands for $|u_{y(x>0)}/u_{y(x<0)}|$ upon the spin-down source. The bars at each frequency are defined as the average ratio plus and/or subtract standard deviation of ratios derived at every group of points, i.e. $|u_{y(x=-30cm)}/u_{y(x=30cm)}|$.

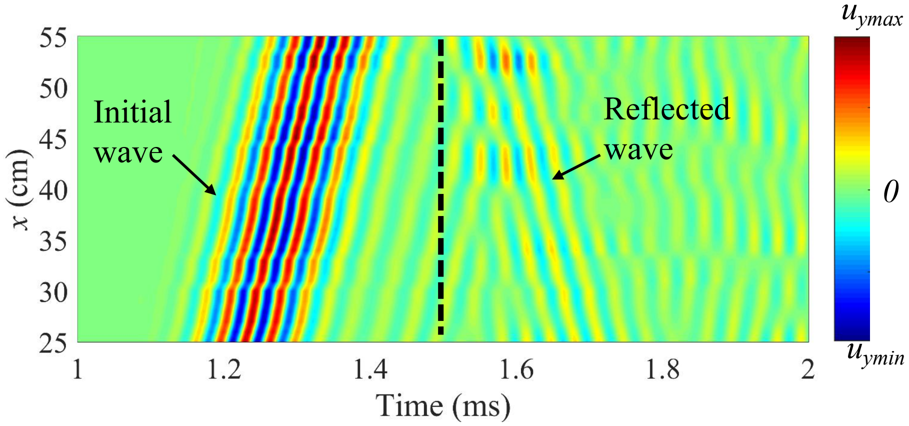

Supplementary Figure 4. The mapping of u_y measured along the segment (x = 25 $\sim$ 55 cm every 1 cm) against time. The propagative initial elastic waves and the reflected elastic waves are distinguished clearly, and the initial waves are not influenced by the reflected ones.

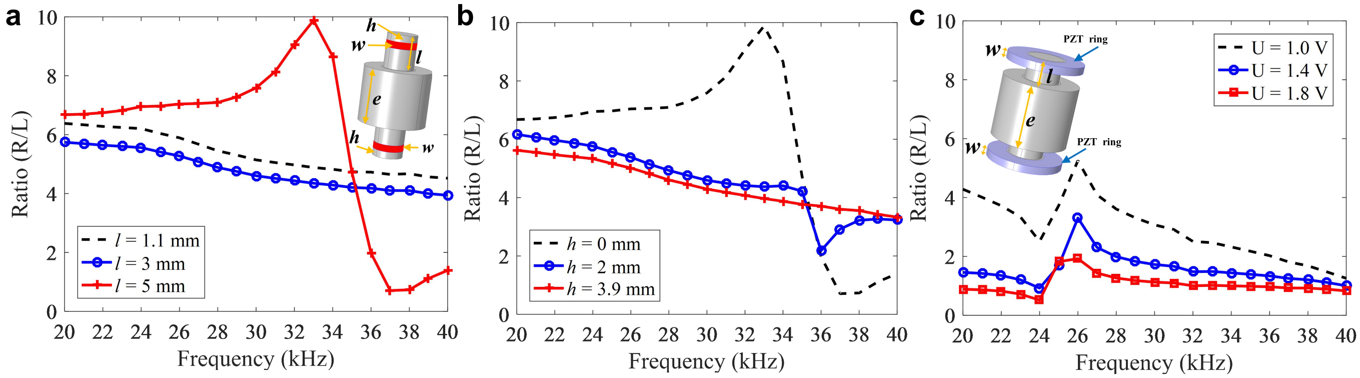

Supplementary Figure 5. **a** The influence of l of symmetrically installed aluminum rods. Uniform pressure with every $\pi/2$ phase delay are applied on ring areas of each rod, shown as the red areas in right-up inset which present one single rod in aluminum plate. Here, we set h = 0, width of ring areas w = 1 mm. **b** The influence of h which represents the position of load areas on installed rods, when l = 5 mm and w = 1. **c** The influence of voltage applied to PZT rings (inner diameter 5 mm, outer diameter 10 mm, thickness 1 mm) installed on rods with l = 3 mm, w = 1 mm, h = 0. Voltage signals with every $\pi/2$ phase delay are applied to each group of PZT rings. The black dashed line is the result when with uniform voltage, 1.0 V, on four group of PZT rings, but the blue circle and red square line show the results when voltage on PZT rings installed on rod 3 are 1.4 and 1.8 times of others.
